# Mapping multidimensional electronic structure and ultrafast dynamics with single-element detection and compressive sensing

**DOI:** 10.1038/ncomms10434

**Published:** 2016-01-25

**Authors:** Austin P. Spencer, Boris Spokoyny, Supratim Ray, Fahad Sarvari, Elad Harel

**Affiliations:** 1 Department of Chemistry, Northwestern University, 2145 Sheridan Road, Evanston, Illinosis 60208, USA

## Abstract

Compressive sensing allows signals to be efficiently captured by exploiting their inherent sparsity. Here we implement sparse sampling to capture the electronic structure and ultrafast dynamics of molecular systems using phase-resolved 2D coherent spectroscopy. Until now, 2D spectroscopy has been hampered by its reliance on array detectors that operate in limited spectral regions. Combining spatial encoding of the nonlinear optical response and rapid signal modulation allows retrieval of state-resolved correlation maps in a photosynthetic protein and carbocyanine dye. We report complete Hadamard reconstruction of the signals and compression factors as high as 10, in good agreement with array-detected spectra. Single-point array reconstruction by spatial encoding (SPARSE) Spectroscopy reduces acquisition times by about an order of magnitude, with further speed improvements enabled by fast scanning of a digital micromirror device. We envision unprecedented applications for coherent spectroscopy using frequency combs and super-continua in diverse spectral regions.

## Abstract (web summary)

Many spectral regions lack the sensitive pixel array detectors needed for multidimensional spectroscopy. Here, the authors report a method for rapidly collecting multidimensional optical spectra without an array detector, instead utilizing spatial encoding and a single-element detector.

## Graphical abstract

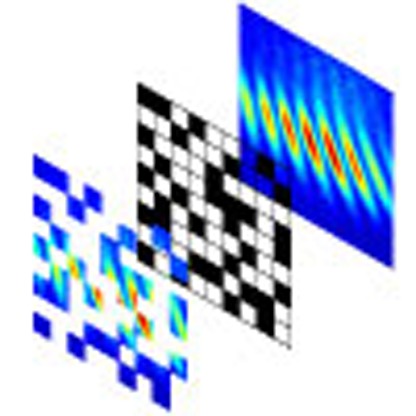

The ability to measure quantum correlations in complex systems with high spectral and temporal resolution provides deep physical insights into a wide range of phenomena from intermolecular dynamics in liquids to the electronic and vibrational structures of condensed phase molecular systems^1^. One powerful approach to directly measure intra- and inter-molecular couplings far from equilibrium is two-dimensional photon echo spectroscopy (2D PES)^2^,^3^,^4^. In 2D PES, coherences encoded in multiple time intervals are correlated, providing insight into the quantum states of the system and their interactions with the surroundings. 2D PES has been used to examine the dynamics of energy transfer in photosynthetic proteins^5^,^6^,^7^, ultrafast dynamics of solute–solvent species^8^ and intraband relaxation in semiconductors^9^,^10^. However, the advantages of performing 2D PES over one-dimensional spectroscopies come at a cost: increased acquisition time, additional experimental complexity and limited sensitivity in regions of the spectrum outside the visible and infrared. Here we introduce a method to overcome these limitations by combining sparse sampling and spatiotemporal encoding of nonlinear optical signals.

To make this connection, we begin by noting that the signal measured in 2D PES is typically sparse (that is, compressible). In most cases, the phase of the nonlinear signal, which is needed to extract the oscillation frequencies of the system, is measured by spectral interferometry in which an external reference field is coherently mixed with the signal and spectrally dispersed onto a detector^11^,^12^,^13^,^14^. Heterodyne detection yields an interferogram that is sparse in one or more Fourier domains, and it is this sparsity that we exploit using compressive sensing (CS) methods.

One consequence of CS theory is effectively to relax the Nyquist–Shannon sampling theorem^15^, which enables perfect reconstruction of continuous signals (as a function of space, time, frequency, and so on) from samples acquired at a rate that is greater than the occupied bandwidth of the signal (irrespective of its distribution) rather than its total bandwidth. In effect, the Nyquist–Shannon sampling theorem relates how to sufficiently sample a signal's frequencies while CS relates how to sufficiently sample a signal's information^16^. CS has been shown to greatly reduce the number of measurements needed for signal recovery for a wide range of applications such as magnetic resonance imaging^17^, nonlinear optical imaging^18^, multidimensional spectroscopy^19^,^20^, holography^21^ and super-resolution microscopy^22^, to name a few. In an analogous way, CS has been used to reduce the number of costly quantum mechanical calculations needed in computational studies^23^,^24^,^25^. One of the most promising applications of CS is in image reconstruction thanks to the potential to (i) utilize single-element detectors in regions of the spectrum where cameras perform poorly and (ii) bypass uniform sampling requirements imposed by array detectors when such sampling is not optimal.

To apply imaging-based CS to coherent spectroscopy, we first need to physically map the 2D spectrum to an image. In 2D PES, the signal is generated by exciting the sample with a series of ultrafast laser pulses, inducing a time-dependent polarization in the sample. The signal is spectrally dispersed onto an array detector, enabling multi-channel acquisition by detecting all signal frequencies, *ω*_*t*_, simultaneously. The experiment is repeated for a range of coherence times, *τ*, and population times, *T*. Fourier transformation along *τ* yields the 2D Fourier transform (2DFT) spectrum *S*(*ω*_*τ*_,*ω*_*t*_;*T*) with parametric dependence on the waiting time, *T*.

Recently, some of the authors introduced a significantly higher-throughput sampling scheme called GRAPES^14^,^26^,^27^ (GRadient Assisted Photon Echo Spectroscopy) that breaks with the traditional *τ*-scan approach. By incorporating a 2D array detector and a spatiotemporal gradient, all the *τ* delays may be sampled in parallel. The signal, emitted from the narrow focal line spatially overlapped with the excitation pulses in the sample, is spectrally resolved along an orthogonal direction, resulting in a direct image of the signal, *I*(*τ*,*ω*_*t*_). Through this imaging arrangement, a 2DFT spectrum is acquired for each laser shot upon Fourier transformation along *τ*. Combining the spatially encoded signal generated in GRAPES with a programmable spatial mask and a single-element detector enables the single-point array reconstruction by spatial encoding (SPARSE) spectroscopy method described here. This application of CS to 2DFT spectroscopy contrasts with prior approaches^19^,^20^ involving sparse sampling of pulse time delays whereby data acquisition time is reduced by sampling a limited window of time delays.

In this work, we present 2DFT spectra of a carbocyanine dye and a photosynthetic pigment-protein complex measured using SPARSE spectroscopy. The accuracy of SPARSE-detected 2DFT spectra is evaluated based on comparison to conventional camera-detected 2DFT spectra. Reconstruction of spatial spectral interferograms is demonstrated using both CS and Hadamard methods, illustrating the robustness of CS retrieval. We show that in some cases, CS reconstruction requires only one-tenth of the complete set of Hadamard-encoded measurements for accurate interferogram recovery.

## Results

### Hadamard encoding and CS reconstruction

**Figure 1:**
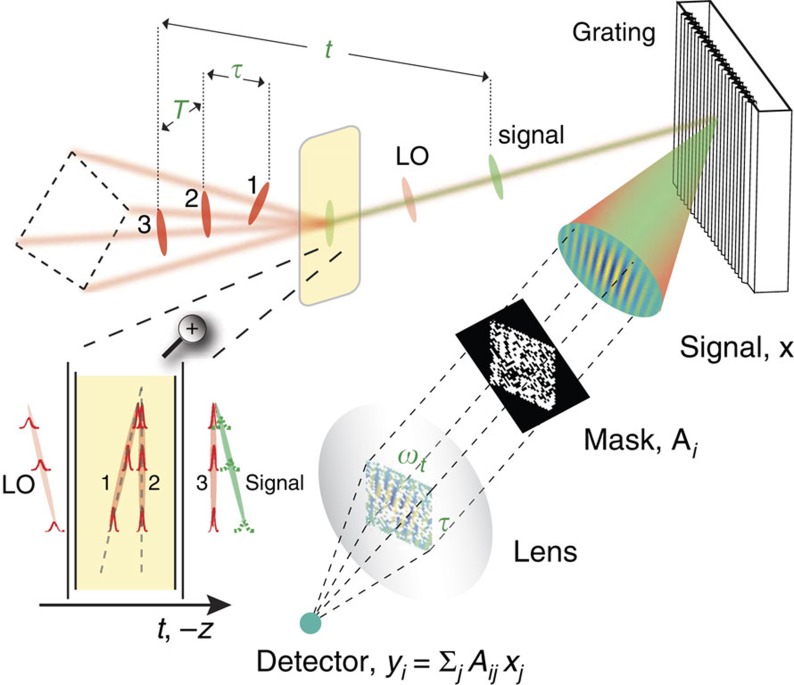
A simplified schematic of single-shot three-pulse photon echo SPARSE spectroscopy. Three pulses (1, 2 and 3) generate a polarization in the sample which subsequently radiates a signal field that spatially interferes with a reference pulse (or local oscillator, LO) at the exit image plane of a spectrometer. The 2D signal-reference interferogram **x**, which spatially encodes the coherence time *τ* (inset shows pulse front tilts of each beam at the sample) and detection frequency *ω*_*t*_, is spatially masked by a DMD (shown as transmissive instead of reflective for simplicity) and then focused by a lens onto a single-element detector. Each mask yields one intensity value on the detector, and by measuring the intensities for a sequence of different masks, the signal-reference interferogram can be retrieved through Hadamard or compressive sensing methods.

**Figure 2:**
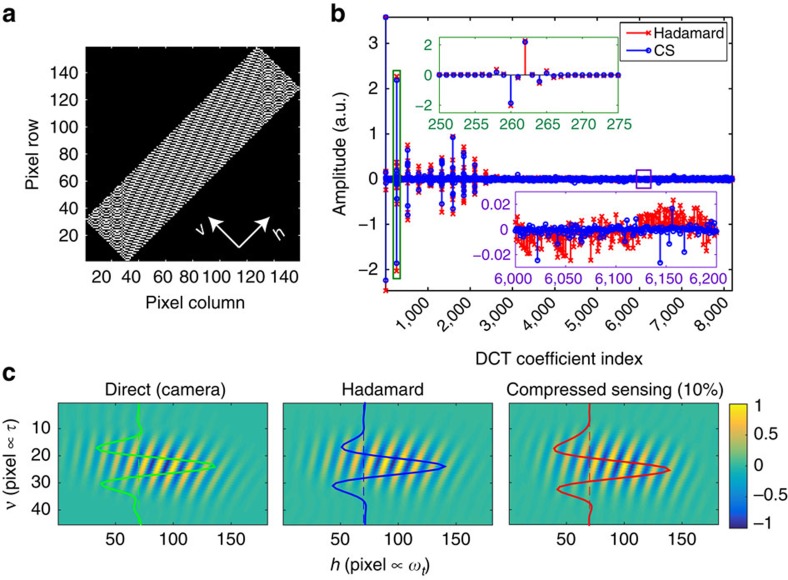
Hadamard encoding and 1D DCT sparsifying transform for the reconstruction of spatial spectral interferograms. (**a**) A single representative Hadamard spatial mask (without random inversions) tiled into the ‘active' region (Methods section) as it appears on the DMD. White arrows indicate horizontal (*h*) and vertical (*v*) lab coordinates. (**b**) Comparison of the 1D DCT of a Hadamard-retrieved flattened interferogram to that recovered by compressive sensing using convex optimization. Insets highlight two regions of the DCT interferogram: one at low spatial frequency (green, upper left) and one at high spatial frequency (purple, lower right). (**c**) Comparison of direct (camera), Hadamard and CS (10 × sub-Nyquist) detected interferograms after Fourier filtering, 45° rotation and cropping. The *v* axis is proportional to the spatially encoded *τ* dimension and the *h* axis is proportional to the detection frequencies, *ω*_*t*_, in the 2DFT spectrum.

To verify the experimental methodology of SPARSE spectroscopy, we first implemented a 2D programmable version of Hadamard spectroscopy^28^,^29^. The Hadamard transform matrix, **H**_*n*_, is analogous to the discrete Fourier transform matrix, but contains only binary (+1 and –1) elements. Hadamard sampling can be expressed as the linear problem **H**_*n*_**x**=**y**, where **x** is a length-*n* signal vector and **y** is a length-*n* measurement vector. The unknown signal **x** can be reconstructed by performing the inverse Hadamard transform on **y**. To implement **H**_*n*_ experimentally, we use a digital micromirror device (DMD)^30^, which is a 2D array of electro-mechanical mirror elements whose surface normal angles can be controlled between two binary states: +12° (‘on' state, 1) and −12° (‘off' state 0; see Methods for details). In this way, multiplication of the masks (each constructed from a different row (*H*_*i*_) of the Hadamard matrix **H**_*n*_) with the unknown image (**x**) occurs optically by reflecting the image formed at the spectrometer exit off of a spatial mask imprinted on the DMD. The reflected portions (that is, pixels) of the image are summed to form the observation 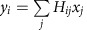 by focusing the reflected light with a lens onto a small active area photomultiplier tube (PMT) detector (Fig. 1). An example spatial mask (without the random inversions described in Method section) is shown in Fig. 2a.

As with the Fourier transform, Hadamard reconstruction requires Nyquist sampling to recover the signal. However, if the signal is sparse under a suitable unitary transform, then according to CS, an *n*-element signal may be faithfully reconstructed from fewer than *n* measurements. CS algorithms (for example, convex optimization and basis pursuit) minimize the L1-norm of the recovered signal subject to the constraint **Ax=y**, where **A** is an *n* × *m* observation matrix and **y** is a length-*m* measurement vector with *m*≤*n*. In this work, **A** is a pseudo-randomly chosen subset of the rows of a Hadamard matrix **H**_*n*_, although other forms are possible such as a random matrix of 0 and 1. It is important to note that **x** itself does not necessarily have to be sparse, but rather it should be sparse in a suitable basis representation. To solve the constrained L1-norm minimization problem we used l_1_-MAGIC, a program that uses standard interior-point methods to solve convex optimization problems^31^.

### Detection schemes

Spatial spectral interferograms of a carbocyanine dye molecule, IR-144 and photosynthetic pigment-protein complex LH2 were acquired with a GRAPES apparatus using two distinct detection schemes: direct detection with a camera and SPARSE detection using a DMD spatial mask and PMT detector. In the direct case, a 2D array detector at the image plane of the spectrometer recorded 2D interferograms with one spatial dimension (along which *τ* is encoded) and one spectral dimension (*λ*∝1/*ω*_*t*_). In the second detection scheme, a DMD placed at the image plane selectively reflects light at each pixel towards (‘on') or away (‘off') from a PMT. A simplified schematic of this apparatus is depicted in Fig. 1. While the camera measures the light intensity incident on each pixel independently, the DMD–PMT detector measures the integrated intensity from all ‘on' DMD pixels. Spatial spectral interferograms for IR-144 detected by both of these methods are shown in Fig. 2c. For SPARSE detection, two reconstruction methods are demonstrated, including reconstruction from a Nyquist-sampled set of Hadamard-encoded observations (Fig. 2c, middle panel) as well as CS reconstruction from a 10 × sub-Nyquist sampled (that is, undersampled) subset of the same Hadamard-encoded measurements (Fig. 2c, right panel). All three interferograms are nearly indistinguishable except for minor differences attributable to noise and reconstruction artifacts in the Hadamard and CS interferograms.

Since heterodyne detection by a time-delayed reference field yields a sinusoidal spectral interference pattern, the discrete cosine transform (DCT) was the natural choice for sparsifying transform. To test the validity of this transform, we explicitly compared the DCT of the CS and Hadamard reconstructed interferograms. To perform the 1D DCT, the interferogram is first flattened to a vector according to the order depicted in Supplementary Fig. 1. The DCT of the interferogram, shown in Fig. 2b, consists mostly of isolated groups of non-zero points. The Hadamard measurement confirms that the DCT is effective at making the signal sparse and also demonstrates good agreement with the CS reconstruction. Notable differences appear in the high frequency DCT coefficients where the relatively constant, low amplitude features in the Hadamard reconstruction contrast with the higher, but less frequent spikes in the CS reconstruction. These deviations are expected due to the inability to exactly reconstruct uncorrelated noise from a sub-Nyquist set of measurements.

### Comparison of 2DFT spectra

**Figure 3:**
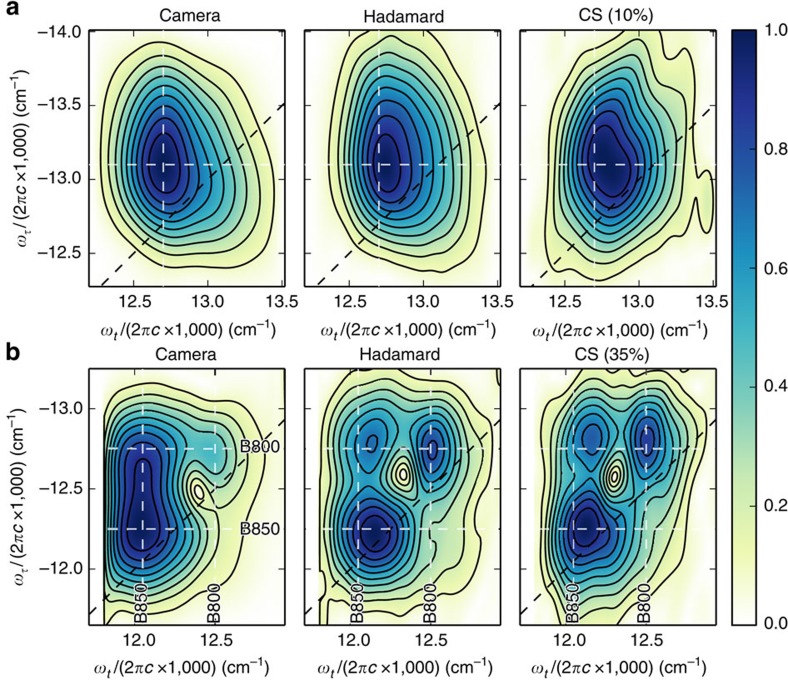
Comparison of 2DFT spectra. Absolute value 2DFT spectra of (**a**) IR-144 cyanine dye (*T*=20 fs) and (**b**) LH2 (*T*=1 ps) detected directly by a camera versus SPARSE (DMD and PMT) detection using either the Hadamard transform (8,192 spatial masks) or compressed sensing with a subset of the Hadamard-encoded measurements (10% (819 spatial masks) for IR-144 and 35% (2,867 spatial masks) for LH2). Diagonal peaks arise from B800 and B850 bands corresponding to the excitation of ring subunits of bacteriochlorophyll pigments in the protein. The upper cross peak results from energy transfer from B800 to B850 in about 1 ps. Approximate locations of band centres are marked with dashed white lines for comparison between figure panels.

Absolute value 2DFT spectra of IR-144 and LH2 in Fig. 3 were constructed (Methods section) from spatial spectral interferograms collected using each of the three detection methods (camera, Hadamard and CS) described above. The Hadamard and CS (10% of Nyquist for IR-144 and 35% for LH2) reconstructions are faithful to the camera-detected spectra with respect to peak positions, although peak shapes and relative amplitudes do differ somewhat, especially in the case of LH2. The fraction of Hadamard-encoded spatial mask measurements used for CS reconstruction of LH2 and IR-144 interferograms was chosen such that variations between 2DFT spectra generated with different pseudo-randomly chosen measurement subsets were below the 10% level. The higher signal-to-noise ratio of IR-144 measurements enabled greater undersampling than for LH2. Statistical variations in CS reconstruction were explored at a range of sampling percentages to ensure that CS reconstructed 2DFT spectra are reproducible and that they converge to the Hadamard reconstruction as sampling approaches 100% (Supplementary Figs 2–15). Some differences between the SPARSE- and camera-acquired 2DFT spectra are expected based on the larger relative decrease in responsivity at longer wavelengths of the PMT compared with the camera. This effect at least partially accounts for the differences in peak amplitudes and, perhaps to a lesser extent, peak shapes between 2DFT spectra acquired using a camera versus a PMT.

While IR-144 (Fig. 3a) has one spectrally broad transition centred ∼800 nm (ref. 32), the spectrum of LH2 contains two absorption bands in the near-infrared, the B800 band at 800 nm (12,500 cm^−1^) and the B850 band at 850 nm (11,765 cm^−1^; ref. 33). Since the laser spectrum is sufficiently broad to excite both transitions, two diagonal peaks are expected at early waiting times (*T*≈0). As the waiting time progresses, molecules initially excited to the higher energy B800 excited state relax to the B850 excited state, producing an off-diagonal cross peak in the spectrum. By *T*=1 ps (Fig. 3b), much of the energy transfer has already taken place, leaving a diagonal peak and a cross peak at the B850 emission frequency and a weak diagonal peak at the B800 emission frequency. These features are readily observed in all three detection modalities.

### Acquisition time comparison

Although a complete set of 8192 Hadamard-encoded spatial mask measurements is acquired in only 0.82 s, the overall speed of the experiment is limited by the need for signal averaging. Despite this, it takes <3 min to acquire the Hadamard-encoded data needed to generate each 2DFT spectra in the middle column of Fig. 3. Taking into account the ability to undersample when using CS reconstruction, this acquisition time can be reduced by a factor ∼3–10. Camera-detected 2DFT spectra, on the other hand, required only about 1 s of acquisition time due to the huge multi-channel advantage afforded by the pixel array sensor. In contrast to SPARSE and GRAPES spectroscopy, 2DFT spectroscopy techniques that involve scanning pulse time delays can take up to 30 min for one scanned axis^3^ or over an hour for two scanned axes^34^. While the most direct comparison for SPARSE spectroscopy would be to phase-sensitive optical detection of 2DFT spectra collected using a single-element detector and scanning both a pulse time delay (*τ*) and a spectral axis (*ω*_*t*_), to our knowledge no such study has been reported. The increase in acquisition speed for SPARSE spectroscopy is afforded by the DMD's ability to perform fast 2D scanning of the GRAPES spatial spectral interferogram image instead of relying on slow scanning of pulse time delays.

## Discussion

To conclude, we demonstrated the use of SPARSE spectroscopy for acquiring 2DFT spectra with sub-Nyquist sampling, reducing by at least an order of magnitude the number of measurements and, consequently, the data collection time. Array detectors impose uniform sampling even when it greatly oversamples the underlying information content of the image. By contrast, the adaptable nature of SPARSE spectroscopy enables, in principle, use of only the minimum number of measurements necessary to sample the information content of the signal given knowledge of the optimum sparsifying transform. While scientific-grade cameras can currently outperform SPARSE in the visible wavelength region thanks to their substantial multi-channel advantage (proportional to the number of pixels), sensitive cameras with high pixel densities are not available in many other spectral regions. Consequently, one of the most exciting applications of this technique is to expand coherent multidimensional spectroscopic methods to the THz and X-ray regimes where nonlinear spectroscopy is extremely challenging, or for use with frequency combs and supercontinuum sources for high-resolution metrology. Overcoming technical hurdles to detection in these spectral regions will be critically important for elucidating fundamental physical phenomena such as the nature of collective and coherent excitations in liquids and atom-specific electronic coherence in transition metal complexes.

## Methods

### Experiment

The 1,028 nm wavelength output of a 306 kHz repetition rate Yb:KGW laser system (PHAROS, Light Conversion) with self-contained oscillator and regenerative amplifier is used to pump a visible-near-infrared noncollinear optical parametric amplifier (ORPHEUS-N, Light Conversion), producing tunable pulses with ∼35 fs duration. The noncollinear optical parametric amplifier output is spatially filtered through a 50-μm diameter pinhole before entering a four-arm interferometer. Four beams, arranged at the corners of a rhombus (or diamond), are focused into the sample cell by a 20-cm focal length cylindrical mirror (refer to Fig. 1 of ref. 14 for details on the beam geometry). The laser centre wavelength was tuned to 775 and 825 nm for measurements on IR-144 and LH2, respectively.

The signal and reference beams are imaged from the sample to the slit of a spectrograph (HR-320, Horiba) by a spherical lens. The spectrograph was modified to create a second output by adding a removable mirror between the second spherical mirror and the standard output. While the standard output houses a CMOS imaging sensor (Zyla 5.5 sCMOS 10-tap, Andor), the added second output is routed to the face of a DMD (DLP9500, Texas Instruments). Since the axes of rotation for the DMD micromirrors lie at a 45° angle relative to the axes of the 1,920 × 1,080 micromirror array, the DMD was rotated by 45° in the plane of its front face so that the reflected light would propagate in a plane parallel to the laser table. The output of the DMD is focused onto a PMT module (H12402, Hamamatsu). The PMT signal is amplified by a fast preamplifier (SR445A, Stanford Research Systems) and then integrated and digitized by a charge-integrating data acquisition (DAQ) system (IQSP518, Vertilon).

The DMD and DAQ are synchronized to the laser pulse train, enabling charge integration of individual laser pulses within a 50 ns window, as well as sub-100 ns control of DMD transition times. The DMD trigger signal, which initiates display of the next available spatial mask in its buffer, was generated by dividing the frequency of the laser clock signal by 32 using a microcontroller (ATmega328, Atmel), ensuring that DMD mask transitions are phase locked to the laser pulse train. In this configuration, each binary mask was held on the DMD for 32 consecutive laser shots, of which 28 laser shots were individually integrated and digitized by the DAQ with the four laser shots closest to the DMD transition being discarded to ensure the measurements are not influenced by motion of the micromirrors. This results in a DMD frame rate of 9.6 kHz, close to its maximum rate of ∼10 kHz. Integrated into the DMD control board is a field-programmable gate array, controller chip, driver and high-capacity on-board memory capable of storing ∼15,000 binary spatial masks.

To perform image reconstruction using a DMD and single-element PMT detector, a set of binary masks was constructed from a 8192^nd^-order (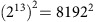 elements) Hadamard matrix whose columns had been randomly inverted. Such inversions make the mask appear spatially quasi-random, which has the benefit of reducing the influence of undesired diffractive contributions to the measured signal that would otherwise arise from variations in diffraction efficiency between different spatial masks. As opposed to full Hadamard multiplexing which requires balanced detection, an S-matrix–like implementation was used here wherein −1 elements of the Hadamard matrix are replaced by 0 such that only +1 elements are detected^29^. To provide adequate spatial resolution while also satisfying the memory limitations of the DMD (see Experimental Limitations and Optimization), the binary mask was limited to an ‘active' region (or region of interest) on the DMD composed of 720 by 180 physical pixels, each containing a single micromirror. Groups of 4 by 4 physical pixels were binned (that is, treated as a single unit) to yield a 180 by 45 region containing 8,100 super-pixels. For each binary mask in the 8,192-frame sequence needed for image reconstruction, a row of the Hadamard matrix was tiled into the 8,100 super-pixel ‘active' region of the DMD (Supplementary Fig. 1); the remainder of the DMD pixels were set to zero and unused Hadamard row elements were discarded. The 45° rotation of the DMD is compensated for by rotating the rectangular bounds of the active region of the spatial mask such that they lie diagonally in the DMD reference frame. This arrangement makes optimal use of the available 8,100 super-pixels since the signals of interest are typically elongated horizontally (in the lab reference frame) by the angular dispersion induced by the spectrograph grating. This rotation is apparent in the spatial mask shown in a since it is plotted in the DMD reference frame.

The sequence of 2^13^=8192 unique DMD frames was repeatedly cycled through until 10–50 million laser shots had been measured, yielding 43–217 complete data sets that can be averaged and reconstructed into an image of the light intensity at the DMD face.

### Data processing

The raw data, each element representing the integrated light intensity of a single laser shot with a given DMD mask, is first divided into complete sequences. Sequences are then averaged together to produce a single 8192-element sequence **y**. To reconstruct the light intensity for each super pixel, the averaged sequence **y** is used to solve the equation **Ax**=**y**, where **A** is the randomly inverted Hadamard matrix used to construct the binary masks and **x** is a vector of super-pixel intensities. Finally, the image at the face of the DMD is obtained by tiling **x** into the active region of the DMD frame exactly as when done to produce the initial DMD masks. An example of an image reconstructed in this way is shown in Fig. 2c (middle panel).

To perform CS reconstruction, first a subset of the Hadamard-encoded measurements described above is pseudo-randomly chosen. The corresponding observation matrix for these measurements is transformed into a sparsifying basis using the 1D DCT along each row. This transformed observation matrix **A** is passed, along with its corresponding intensity measurements **y**, to a convex optimization routine that minimizes the L1-norm of the solution **x** subject to the constraint **Ax**=**y**. The solution is inverse transformed using the 1D DCT to yield the pixel intensity values, which are subsequently tiled into the appropriate spatial arrangement as described above (Supplementary Fig. 1).

2DFT spectra are generated in the same way for both Hadamard and CS reconstructed interferograms. Interferograms first undergo a coordinate transform (involving a 45° rotation with cubic interpolation) from the DMD coordinate space to the camera coordinate space such that a pixel at a given wavelength and *τ* on the DMD maps to the equivalent wavelength and ***τ*** pixel position on the camera. This allows SPARSE-detected and camera-detected interferograms to be processed identically and to share the same pulse delay calibration information. Transformed interferograms are subsequently cropped to the extent of the active area of the DMD spatial mask. The ***τ*** axis is calibrated by measuring the spatial spectral interference between beams 1 and 2 using the spectrograph camera, as previously described^14^. The signal-beam 4 interferograms are (piecewise cubic) interpolated from a grid of equidistant wavelengths to a grid of equidistant frequencies. The interpolated interferograms are 2D Fourier transformed and filtered in the conjugate domain to select one of the two AC interference peaks. A 1D inverse Fourier transform along the *t* dimension (that is, the detection axis) then yields a distorted 2DFT spectrum which must be modified to correct for the crossing angle-induced wavefront tilt between the reference beam (beam 4) and beam 3, as previously reported^14^. Finally, the corrected 2DFT spectra are linearly interpolated to equalize the frequency grid spacing along both the *ω*_*τ*_ and *ω*_*t*_ dimensions. Camera-acquired interferograms are processed identically save for the initial coordinate transform. The camera-acquired interferograms are cropped to the extent of the DMD active area for comparison to SPARSE detection.

### Experimental limitations and optimization

There are some properties of the experimental apparatus and methods presented here that are thought to limit the accuracy of 2DFT spectra collected using the implementation of SPARSE detection described here. First, collecting signal from a limited spatial area of the DMD causes the very low-lying wings of the signal to be lost (Fig. 2c). This limitation arises from the finite size of the DMD's on-board memory which, in turn, limits the number of unique DMD spatial masks that can be loaded at a given time. Since the number of pixels in a recovered interferogram image is related to the number of DMD spatial mask measurements, for a fixed number of measurements, there exists a tradeoff between an image's spatial resolution and its size. The spatial size of features in the interferogram image (for example, interference fringes, spectral lineshapes and transient dynamics) sets the minimum required spatial resolution. This spatial resolution requirement, combined with the limit in number of measurements, sets the maximum spatial extent of the region able to be sampled on the DMD. For Hadamard reconstruction, the on-board memory limits the number of recovered image pixel to ∼15,000 since reconstruction of an *n* pixel image requires measurement of *n* unique DMD spatial masks. The 720 by 180 physical pixel region used corresponds to a 7.8 mm by 1.9 mm area, less than a quarter of the 16.6 mm by 4.6 mm area collected by the camera. This restriction could be alleviated either by increasing the on-board memory or by using CS reconstruction with ≤15,000 DMD spatial mask chosen randomly from a larger Hadamard matrix (that is, **H**_*n*_ where *n*>15,000).

Second, the small active area of the PMT (3 mm by 1 mm) requires careful alignment to ensure that all light reflected from the DMD is collected to avoid attenuating the edges of the interferogram. In addition, the small active area limits the maximum permissible energy per pulse incident on the detector to maintain detector linearity, which is critical for accurate interferogram reconstruction. To avoid detector saturation, likely due to space charge effects^35^, lower than optimal reference beam pulse energies were used, limiting the heterodyne advantage of spectral interferometry.

Third, the measured signal is weighted by the spectral response of the PMT, which decreases at longer wavelengths. Although the same is true for the CMOS camera used here, the change in response is much larger for the PMT. Moving from 850 to 800 nm, the spectral response of the PMT increases by a factor of ∼3 compared with a factor of ∼1.5 increase for the camera. Overall, the PMT has a low quantum efficiency (∼0.5%, compared with ∼28% for the camera) which somewhat limits the signal-to-noise ratio of SPARSE detection. Signal-to-noise ratio could be improved by using a higher quantum efficiency detector such as an avalanche photodiode.

## Additional information

**How to cite this article:** Spencer, A. P. *et al*. Mapping multidimensional electronic structure and ultrafast dynamics with single-element detection and compressive sensing. *Nat. Commun.* 7:10434 doi: 10.1038/ncomms10434 (2016).

## Supplementary Material

Supplementary Information Supplementary Figures 1-15
