# Supplementary material for: Mapping multidimensional electronic structure and ultrafast dynamics with single-element detection and compressive sensing
Source: Nat Commun. 2016 Jan 25;7:10434. doi: 10.1038/ncomms10434 (PMC4737750; doi:10.1038/ncomms10434)
Supplement: Supplementary Information — Supplementary Figures 1-15 [file ncomms10434-s1.pdf]

SUPPLEMENTARY INFORMATION

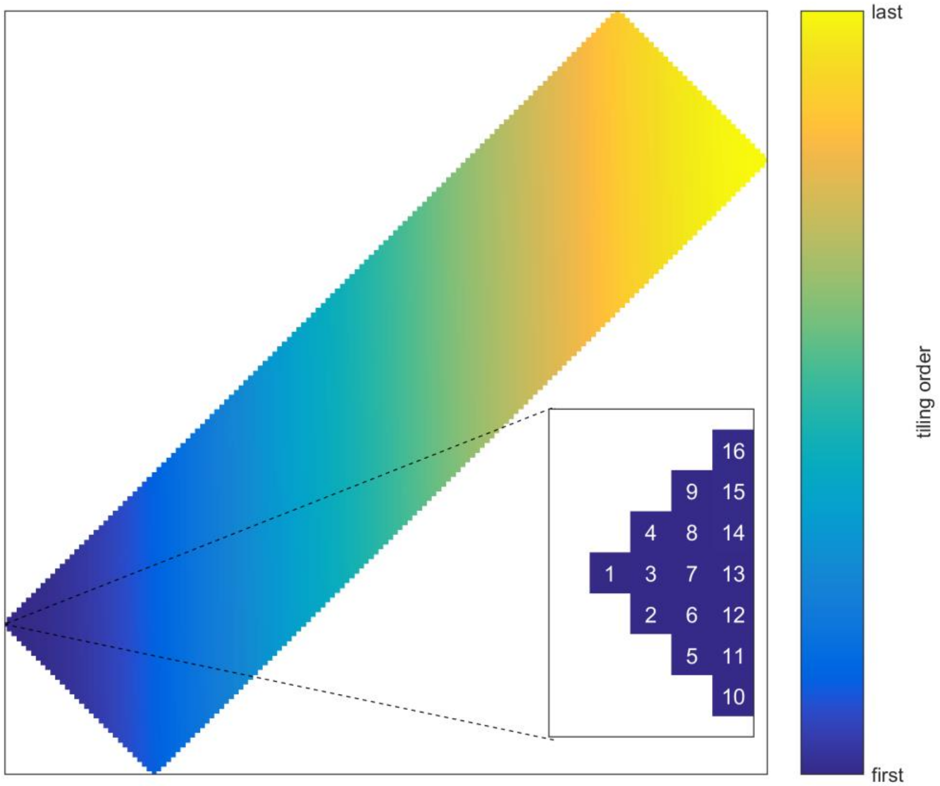

Supplementary Figure 1. Spatial mask tiling order. Illustration of the tiling order of a single row of a Hadamard matrix into a DMD spatial mask. In the inset, numbers indicate the order of the first 16 Hadamard elements tiled (or wrapped) into the active region. The pixels outside of the active region are shown in white.

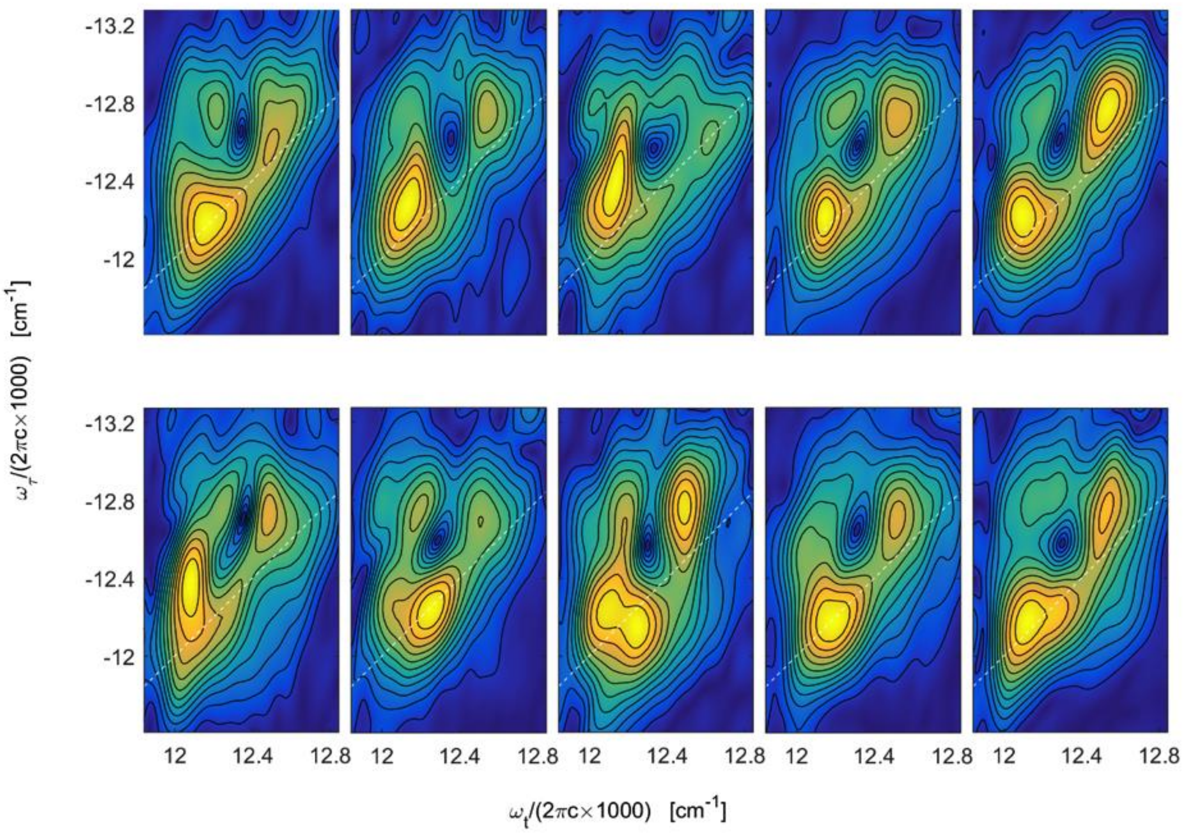

Supplementary Figure 2. 2DFT spectra of LH2 with 15% sampling. Absolute value 2DFT spectra of LH2 generated from CS reconstructed interferograms using 1229 pseudo-randomly chosen measurements/spatial masks, 15% of the complete set of 8192 Hadamard-encoded spatial mask measurements. The 2DFT spectrum in each panel is generated from a different random set of 1229 measurements/spatial masks.

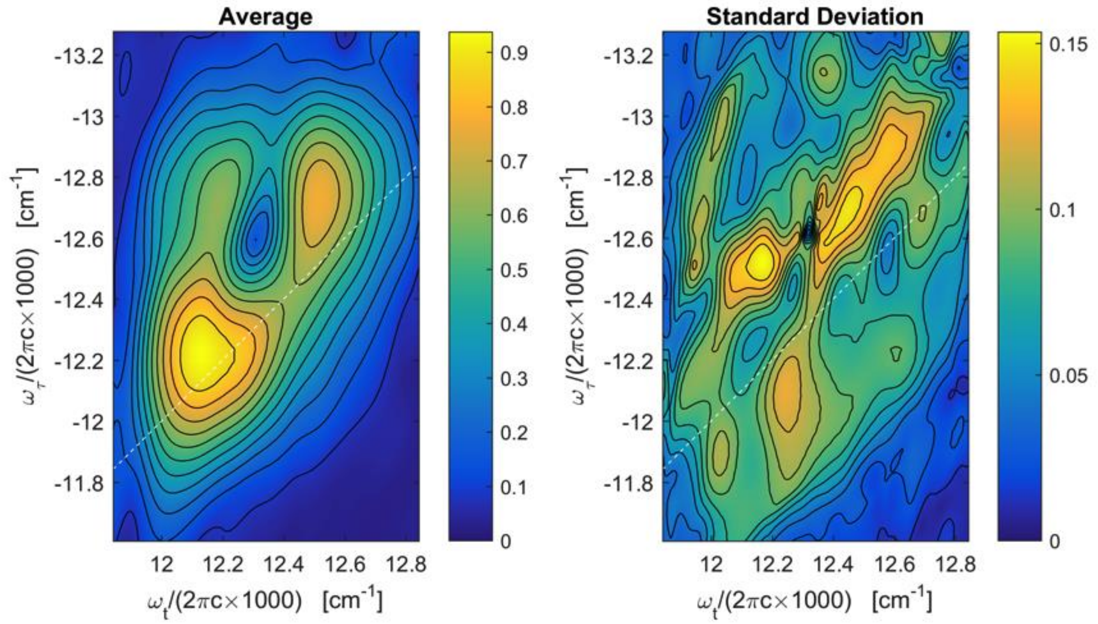

Supplementary Figure 3. Statistics for 2DFT spectra of LH2 with 15% sampling. The average and standard deviation of the 10 absolute value 2DFT spectra of LH2 shown in Supplementary Figure 2 generated from CS reconstructed interferograms using 15% of the complete set of Hadamard-encoded spatial mask measurements.

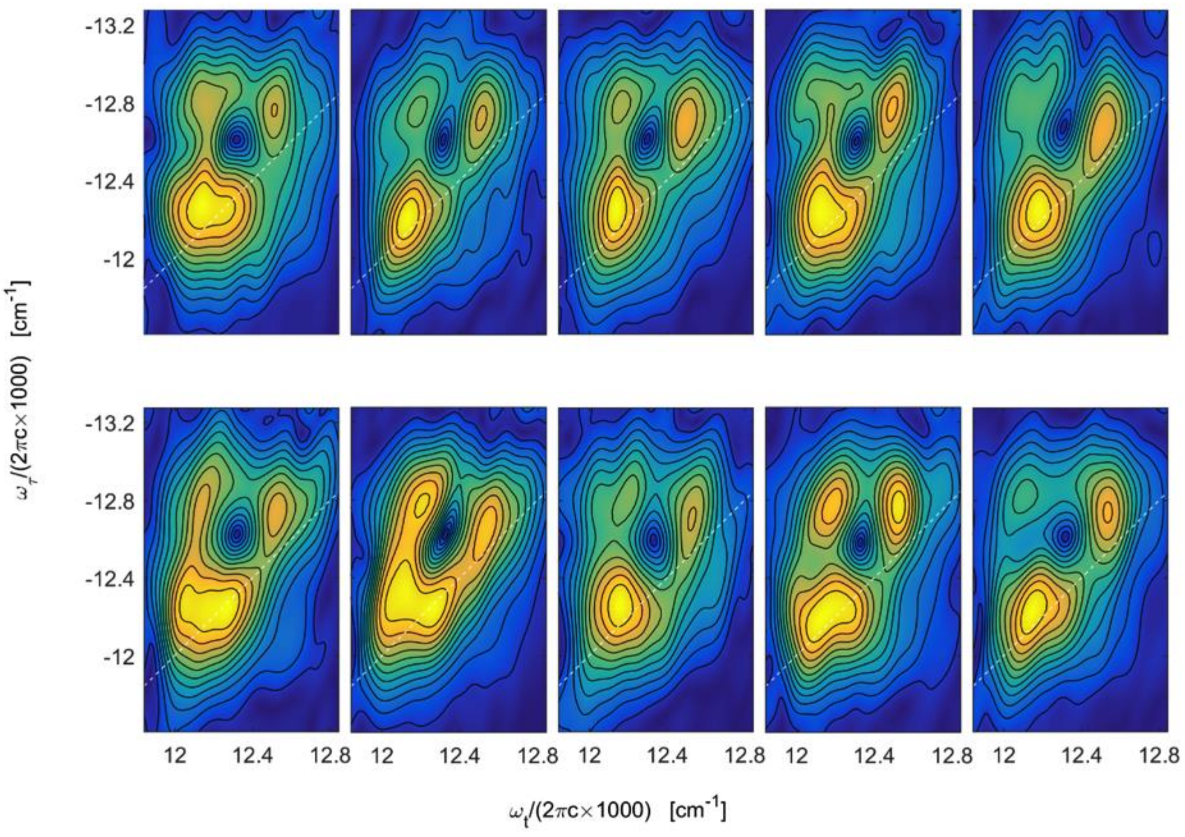

Supplementary Figure 4. 2DFT spectra of LH2 with 25% sampling. Absolute value 2DFT spectra of LH2 generated from CS reconstructed interferograms using 2048 pseudo-randomly chosen measurements/spatial masks, 25% of the complete set of 8192 Hadamard-encoded spatial mask measurements. The 2DFT spectrum in each panel is generated from a different random set of 2048 measurements/spatial masks.

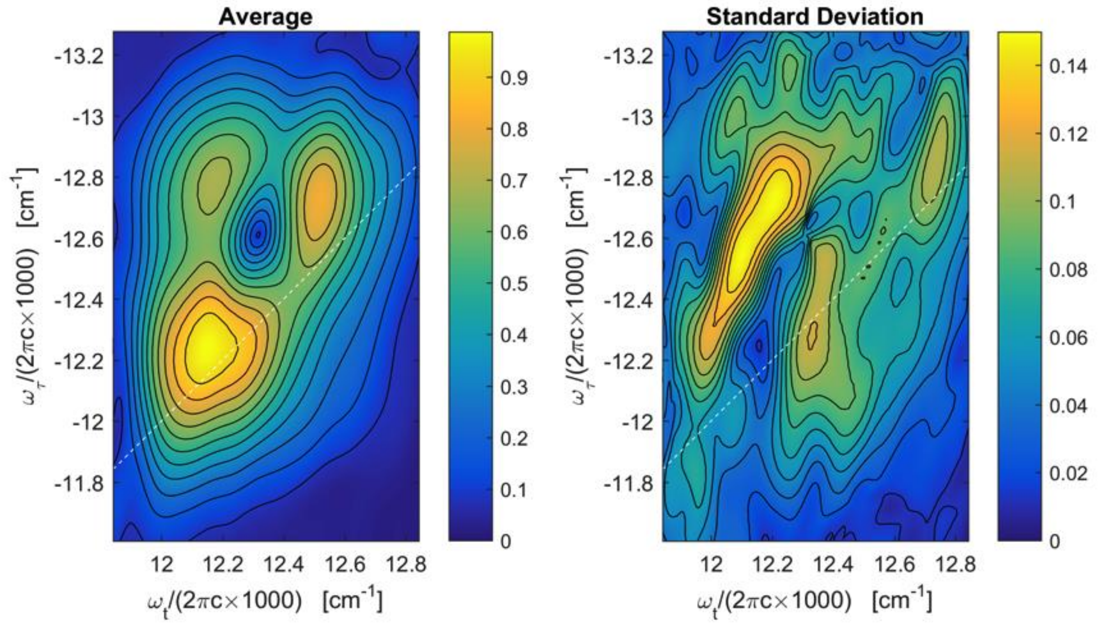

Supplementary Figure 5. Statistics for 2DFT spectra of LH2 with 25% sampling. The average and standard deviation of the 10 absolute value 2DFT spectra of LH2 shown in Supplementary Figure 4 generated from CS reconstructed interferograms using 25% of the complete set of Hadamard-encoded spatial mask measurements.

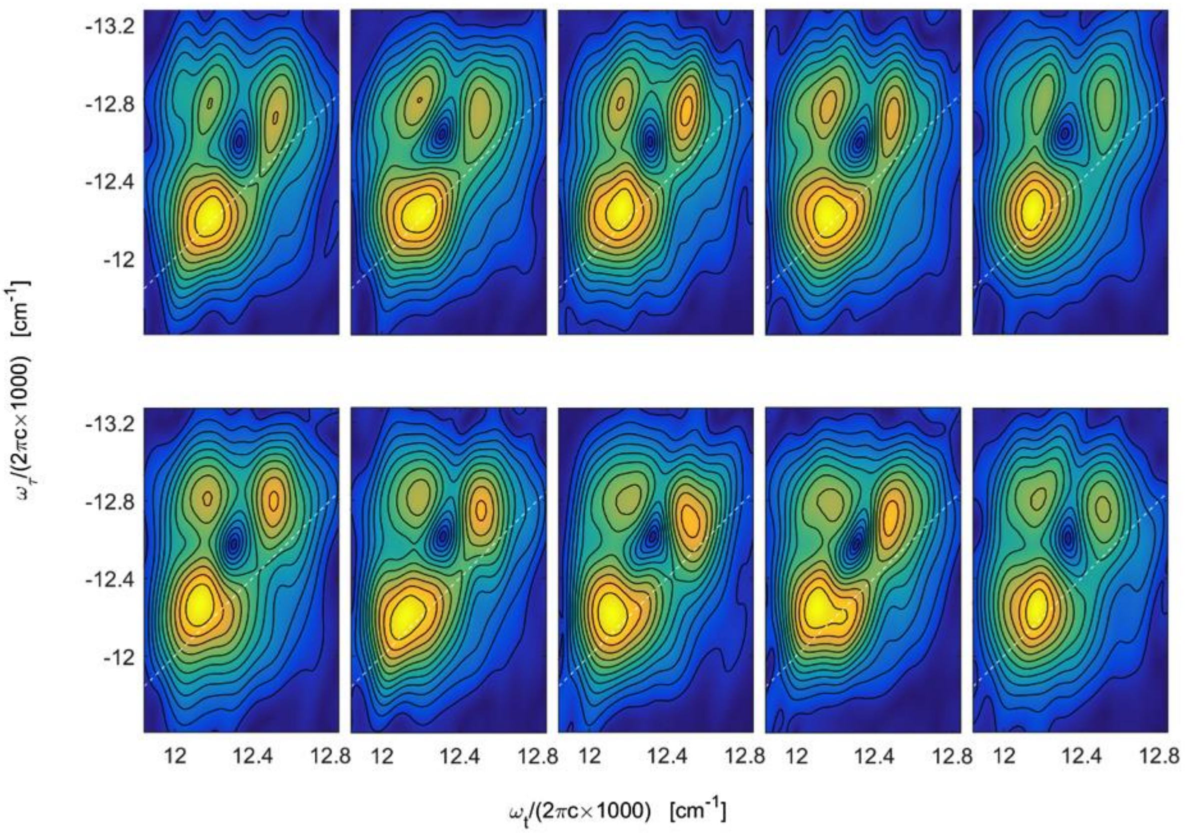

Supplementary Figure 6. 2DFT spectra of LH2 with 35% sampling. Absolute value 2DFT spectra of LH2 generated from CS reconstructed interferograms using 2867 pseudo-randomly chosen measurements/spatial masks, 35% of the complete set of 8192 Hadamard-encoded spatial mask measurements. The 2DFT spectrum in each panel is generated from a different random set of 2867 measurements/spatial masks.

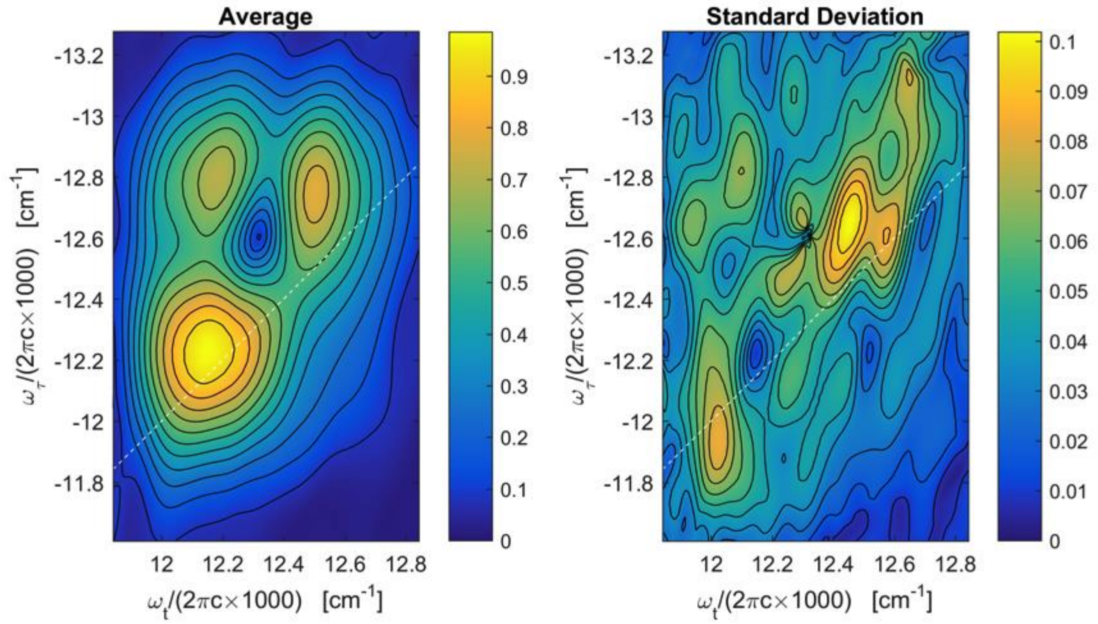

Supplementary Figure 7. Statistics for 2DFT spectra of LH2 with 35% sampling. The average and standard deviation of the 10 absolute value 2DFT spectra of LH2 shown in Supplementary Figure 6 generated from CS reconstructed interferograms using 35% of the complete set of Hadamard-encoded spatial mask measurements.

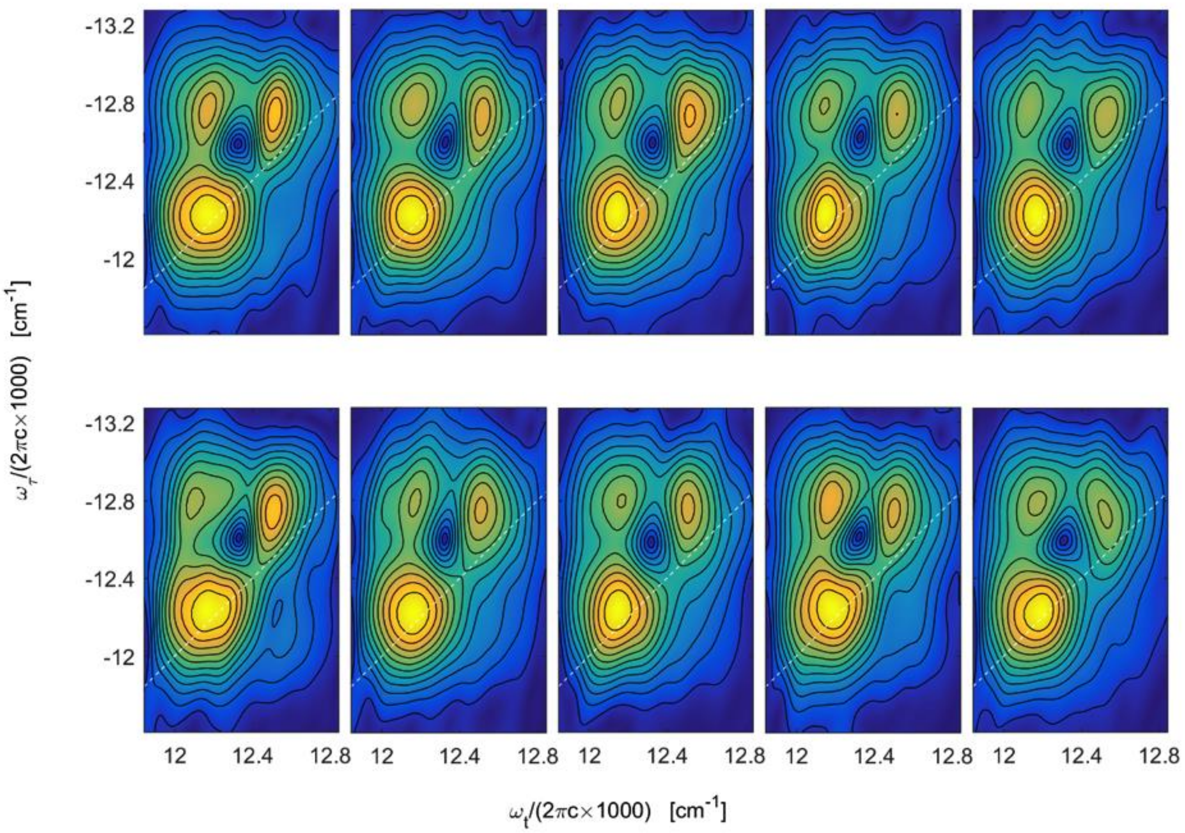

Supplementary Figure 8. 2DFT spectra of LH2 with 50% sampling. Absolute value 2DFT spectra of LH2 generated from CS reconstructed interferograms using 4096 pseudo-randomly chosen measurements/spatial masks, 50% of the complete set of 8192 Hadamard-encoded spatial mask measurements. The 2DFT spectrum in each panel is generated from a different random set of 4096 measurements/spatial masks.

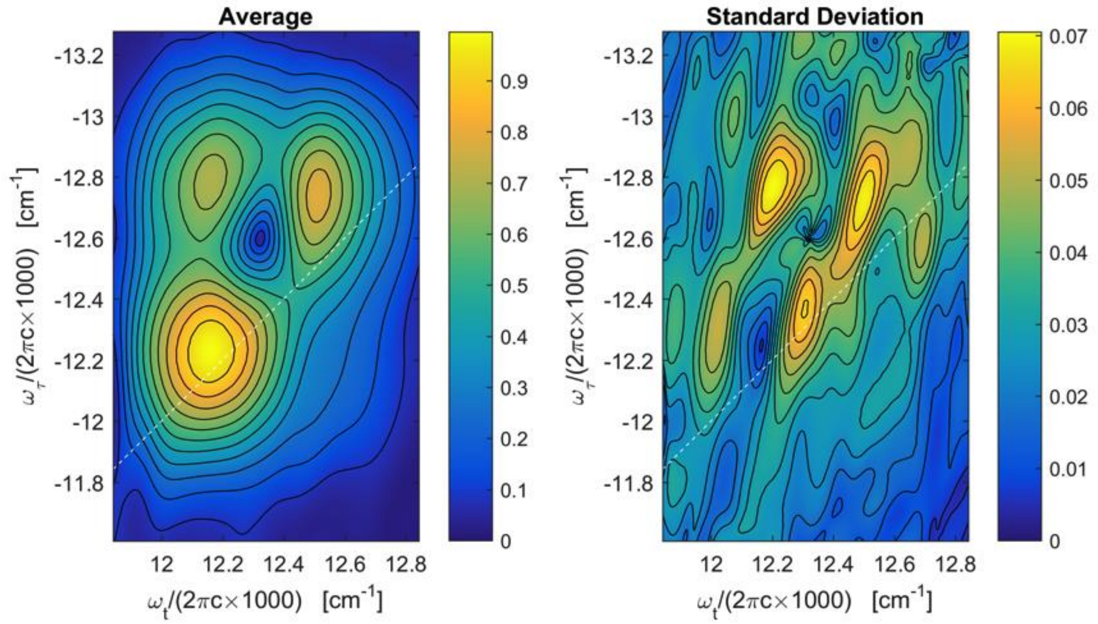

Supplementary Figure 9. Statistics for 2DFT spectra of LH2 with 50% sampling. The average and standard deviation of the 10 absolute value 2DFT spectra of LH2 shown in Supplementary Figure 8 generated from CS reconstructed interferograms using 50% of the complete set of Hadamard-encoded spatial mask measurements.

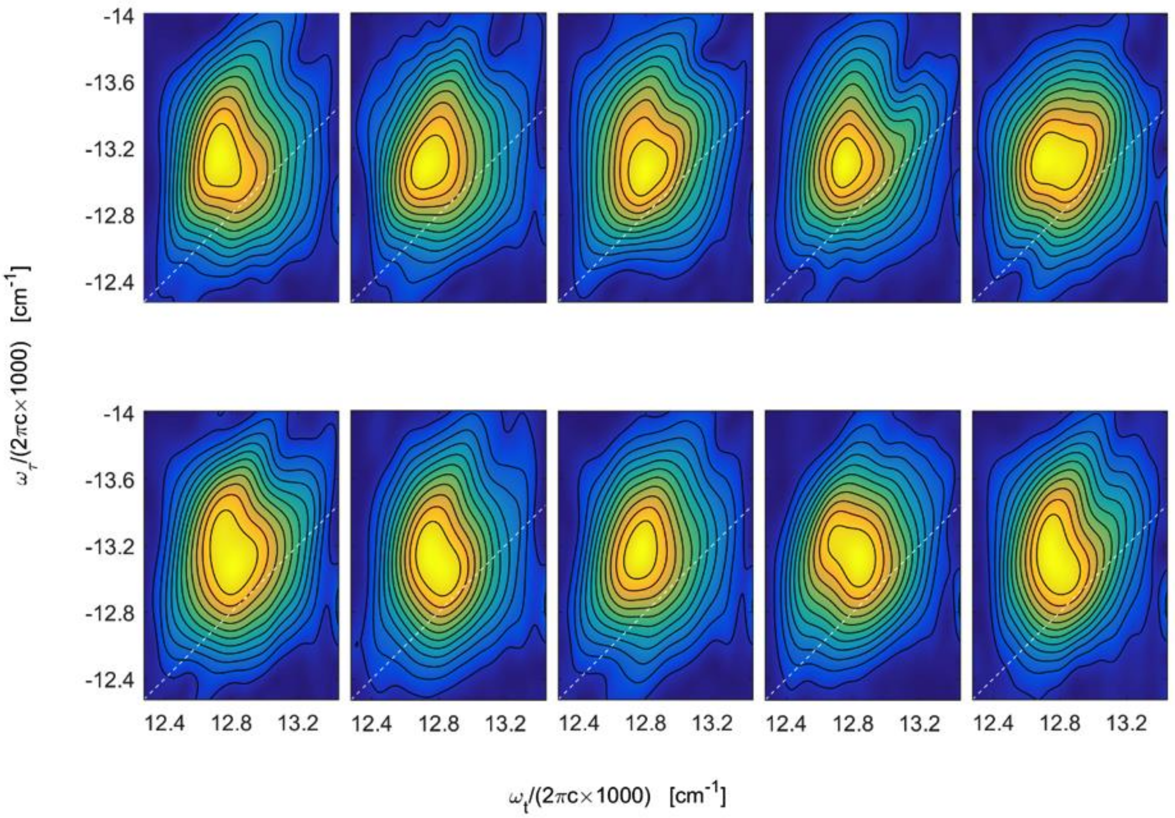

Supplementary Figure 10. 2DFT spectra of IR-144 with 10% sampling. Absolute value 2DFT spectra of IR-144 generated from CS reconstructed interferograms using 819 pseudo-randomly chosen measurements/spatial masks, 10% of the complete set of 8192 Hadamard-encoded spatial mask measurements. The 2DFT spectrum in each panel is generated from a different random set of 819 measurements/spatial masks.

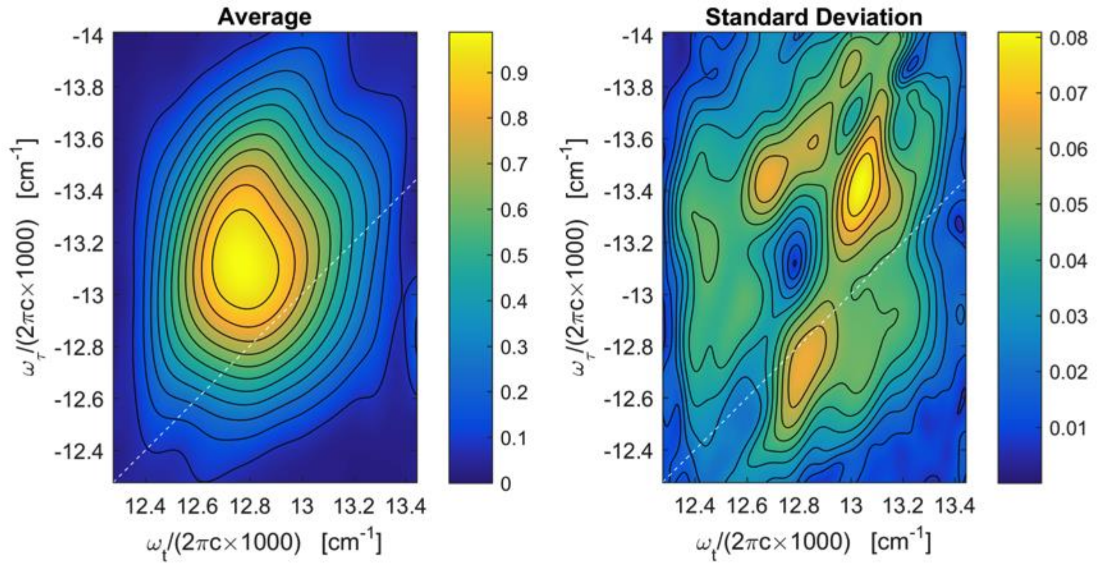

Supplementary Figure 11. Statistics for 2DFT spectra of IR-144 with 10% sampling. The average and standard deviation of the 10 absolute value 2DFT spectra of IR-144 shown in Supplementary Figure 10 generated from CS reconstructed interferograms using 10% of the complete set of Hadamard-encoded spatial mask measurements.

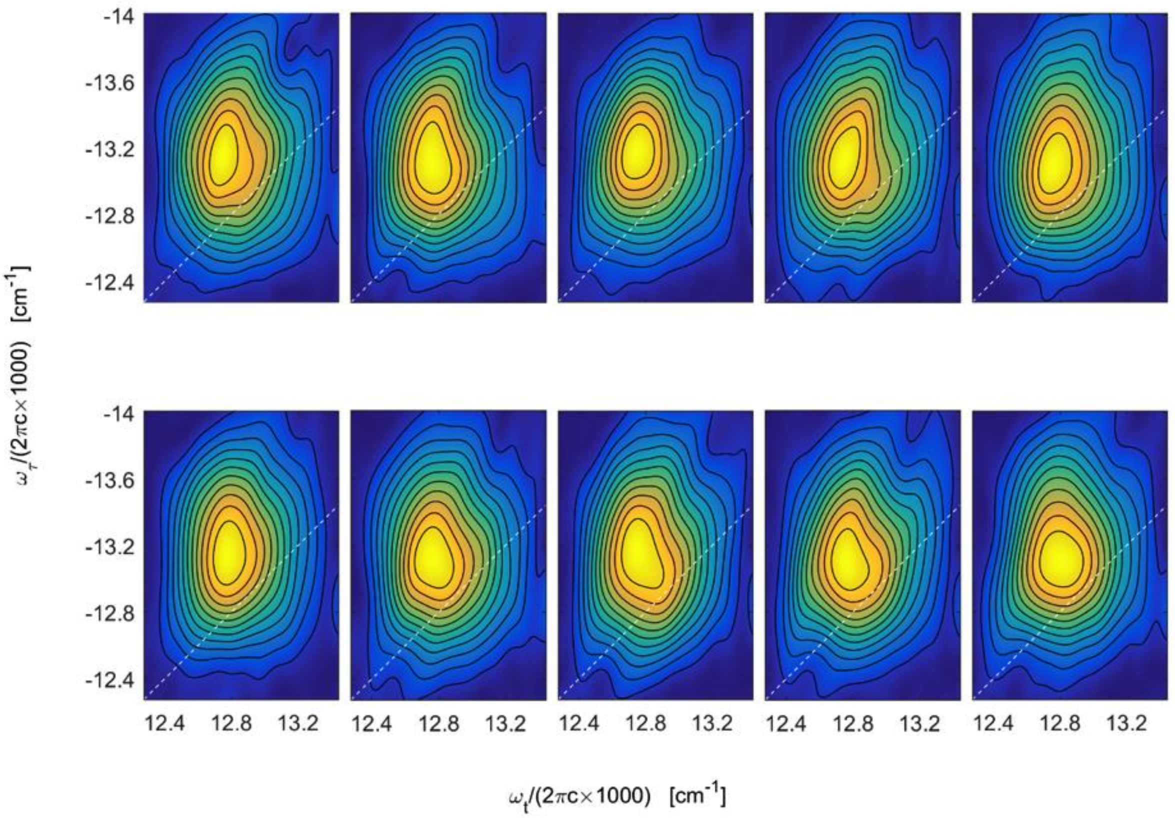

Supplementary Figure 12. 2DFT spectra of IR-144 with 15% sampling. Absolute value 2DFT spectra of IR-144 generated from CS reconstructed interferograms using 1229 pseudo-randomly chosen measurements/spatial masks, 15% of the complete set of 8192 Hadamard-encoded spatial mask measurements. The 2DFT spectrum in each panel is generated from a different random set of 1229 measurements/spatial masks.

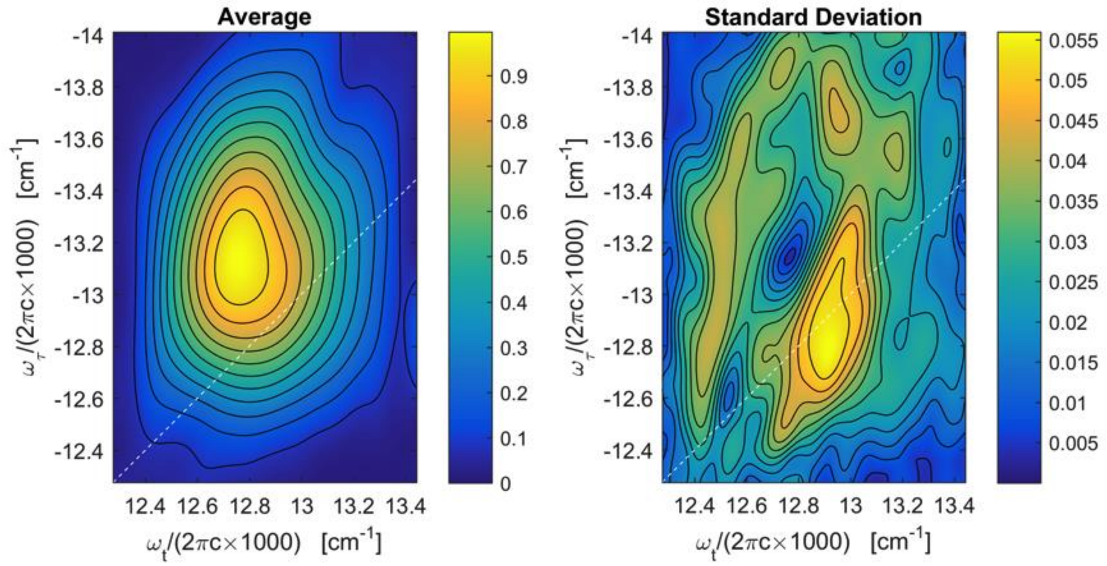

Supplementary Figure 13. Statistics for 2DFT spectra of IR-144 with 15% sampling. The average and standard deviation of the 10 absolute value 2DFT spectra of IR-144 shown in Supplementary Figure 12 generated from CS reconstructed interferograms using 15% of the complete set of Hadamard-encoded spatial mask measurements.

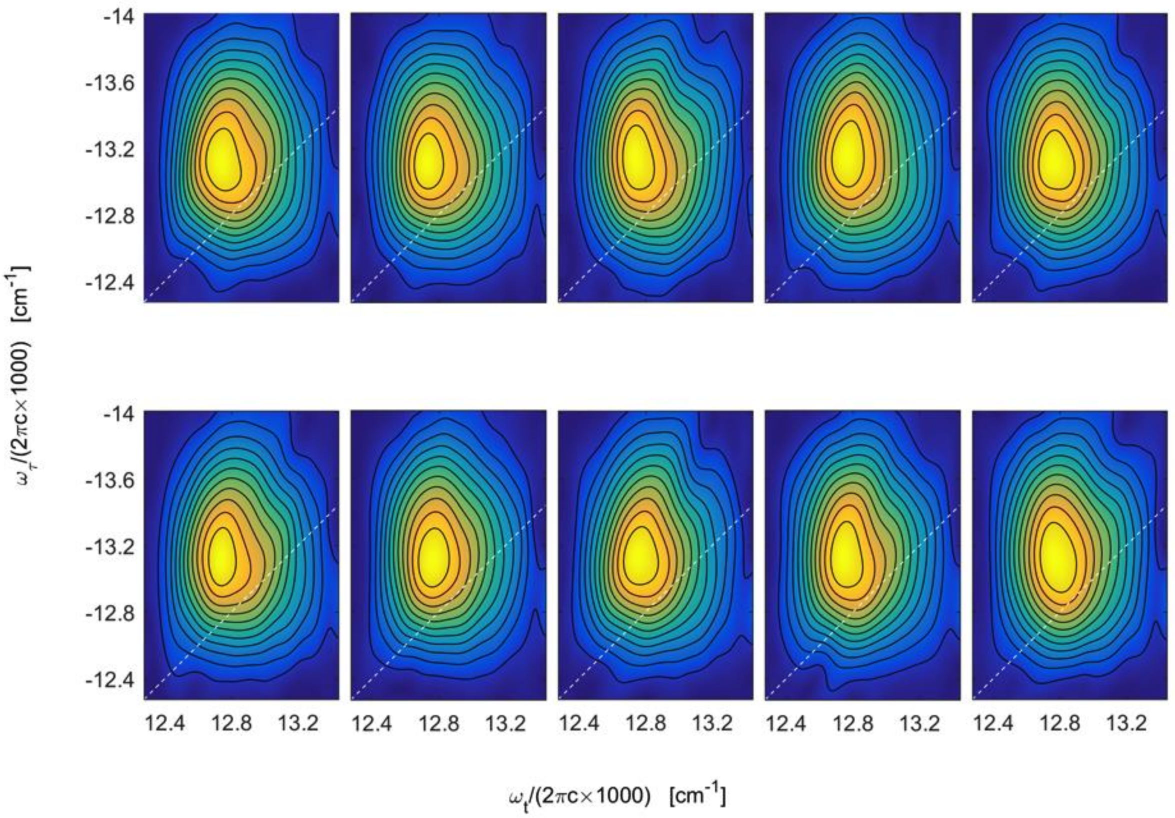

Supplementary Figure 14. 2DFT spectra of IR-144 with 25% sampling. Absolute value 2DFT spectra of IR-144 generated from CS reconstructed interferograms using 2048 pseudo-randomly chosen measurements/spatial masks, 25% of the complete set of 8192 Hadamard-encoded spatial mask measurements. The 2DFT spectrum in each panel is generated from a different random set of 2048 measurements/spatial masks.

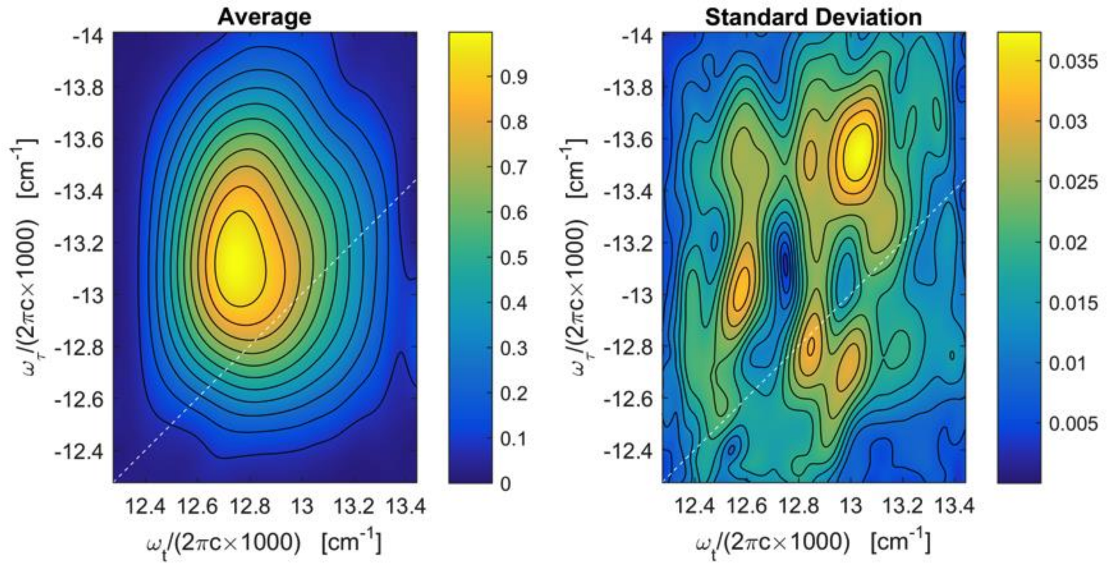

Supplementary Figure 15. Statistics for 2DFT spectra of IR-144 with 25% sampling. The average and standard deviation of the 10 absolute value 2DFT spectra of IR-144 shown in Supplementary Figure 14 generated from CS reconstructed interferograms using 25% of the complete set of Hadamard-encoded spatial mask measurements.
